# Retrospective study on the value of serum angiopoietin 2 and cystatin C levels in the early diagnosis of acute coronary syndrome

**DOI:** 10.1097/MD.0000000000043650

**Published:** 2025-08-15

**Authors:** Qian Zou, Yuxia Huang, Yabin Peng, Xuanhao Lu, Feilin Ye, Jie Xu, Ling Fang

**Affiliations:** a Department of Emergency, Air Force 986th Hospital, Xi’an, China; b Department of Outpatient, Air Force 986th Hospital, Xi’an, China; c Department of Cardiology, Air Force 986th Hospital, Xi’an, China; d Department of Cardiology, Yichang Central People’s Hospital, Yichang, China; e The First College of Clinical Medical Science, China Three Gorges University, Yichang, China.

**Keywords:** acute coronary syndrome, Ang-2, Cys-C, early diagnosis, independent risk factor, ROC

## Abstract

Acute coronary syndrome (ACS) is a coronary emergency that arises from myocardial ischemia and thrombosis and can be triggered by the rupture of a subcutaneous unstable plaque within the coronary artery or coronary artery erosion. The current study aimed to calculate the predictive value of serum angiopoietin 2 (Ang-2) and cystatin C (Cys-C) levels in the early diagnosis of ACS. We retrospectively analyzed data from 180 patients diagnosed with ACS at our hospital between January 2023 and June 2024, with 120 healthy volunteers serving as the control group during the same period. Clinical baseline and pathological data were recorded for all participants, and serum levels of Ang-2 and Cys-C were determined using an enzyme-linked immunosorbent assay kit. The correlation between serum Ang-2, Cys-C levels, and Gensini scores in patients with ACS was analyzed using Spearman or Pearson correlation coefficients, respectively. Independent risk factors for ACS were analyzed using multivariate logistic regression. A receiver operating characteristic curve was used to analyze the predictive value of serum Ang-2, Cys-C, or Ang-2 combined with Cys-C for the early diagnosis of ACS. Serum Ang-2 and Cys-C levels in patients with ACS were significantly higher than those in the normal group. Serum Ang-2 and Cys-C levels significantly and positively correlated with Gensini scores in patients with ACS. Logistic multivariate regression analysis revealed that total cholesterol, triglycerides, low-density lipoprotein cholesterol, Ang-2, and Cys-C were independent risk factors for ACS. The area under the curve of serum Ang-2 combined with Cys-C was 0.897 (sensitivity, 77.22%; specificity, 87.50%) in patients with ACS, and its diagnostic efficacy was higher than that of Ang-2 or Cys-C alone. Serum Ang-2 and Cys-C are highly expressed in patients with ACS, and serum Ang-2 combined with Cys-C has a high predictive value for the early diagnosis of ACS.

## 1. Introduction

Acute coronary syndrome (ACS) is a coronary emergency that arises from myocardial ischemia and thrombosis and can be triggered by the rupture of a subcutaneous unstable plaque within the coronary artery or coronary artery erosion. The incidence of this condition increases every year; it has a rapid onset, with high mortality rates.^[1]^ The clinical diagnosis of ACS is usually based on clinical symptoms, an electrocardiogram, and elevated cardiac troponin T (cTnT). However, a large number of patients with ACS are still misdiagnosed, and even 2% to 5% of patients are missed in the emergency department.^[2,3]^ Currently, cTnT or creatine kinase isoenzyme (CK-MB) is used as a cardiac biomarker to evaluate ACS; however, the sensitivity of early diagnosis of ACS in clinical practice is not high, with certain limitations.^[4,5]^ Therefore, the search for convenient and sensitive biomarkers is vital for the diagnosis and effective treatment of ACS.

Angiopoietin 2 (Ang-2) is a cellular regulatory factor that acts on vascular endothelial cells and plays an important role in the process of new angiogenesis.^[6]^ Ang-2 levels are elevated in a rat model of myocardial infarction.^[7]^ It is highly expressed in the myocardial tissue of patients with myocardial ischemia.^[8]^ In addition, there have been reports of elevated plasma Ang-2 levels in patients with ACS.^[9]^

Cystatin C (Cys-C) is a cysteine protease inhibitor, and higher levels of Cys-C are correlated with the severity of coronary atherosclerosis or coronary heart disease.^[10,11]^ Increased Cys-C levels in patients with ACS are associated with increased mortality.^[12]^ Serum levels of Cys-C are significantly elevated in patients with non-ST-elevation ACSs (NSTE-ACS). Cys-C can be an independent predictor of major adverse cardiac events.^[13]^ However, the diagnostic value of combining serum Ang-2 and Cys-C levels in the early detection of ACS remains uncertain.

While previous studies have separately explored the relationship between Ang-2 and Cys-C in cardiovascular diseases, most have focused only on the impact of a single-marker or have not prioritized the early diagnosis of ACS. This study is the first to combine Ang-2 and Cys-C levels to systematically evaluate their potential value in the early diagnosis of ACS. Through multivariate analysis, we confirmed the significant role of these 2 markers as independent risk factors for ACS and compared the diagnostic performance of combined detection and single-marker detection using receiver operating characteristic (ROC) curves. This study offers a novel perspective and a promising biomarker combination for the early diagnosis of ACS, which has not been reported in prior studies. Given the lack of a comprehensive evaluation of these biomarkers in combination for ACS diagnosis, this study aimed to explore the value of serum Ang-2 combined with Cys-C levels in the early diagnosis of ACS and to provide important clinical value for the early diagnosis and treatment of ACS.

## 2. Materials and methods

### 2.1. Study participants

This study retrospectively analyzed 230 patients diagnosed with ACS in our hospital from January 2023 to June 2024, of whom 41 did not meet the inclusion criteria, and 9 had incomplete data; hence, 180 patients with ACS were included in the final analysis. All participants were informed of the purpose of the study and signed an informed consent form, which was reviewed and approved by the Ethics Committee of the Yichang Central People’s Hospital Institute following the Declaration of Helsinki.

The inclusion criteria^[14]^ were as follows: patients who met the diagnostic criteria of the Guidelines for Rapid Emergency Diagnosis and Treatment of Acute Coronary Syndrome (2019); all patients with ACS who experienced their first attack; all enrolled patients who underwent coronary angiography examination; all enrolled patients aged 45 to 80 years old; and patients who had complete clinical and laboratory data.

The exclusion criteria^[14,15]^ were as follows: Patients with malignant arrhythmia, coarctation of the aorta, heart failure, cardiomyopathy or severe valvular disease; accompanied by serious liver and kidney dysfunction, infectious diseases, or malignant tumors; patients with a history of cardiac stent implantation or coronary artery bypass grafting; pregnant women and lactating mothers; patients with autoimmune diseases in an active stage (evidenced by elevated autoantibody levels, ongoing disease-specific symptoms, and need for immunosuppressive treatment), as these may interfere with the levels of Ang-2 and Cys-C through immune-mediated mechanisms; patients taking medications known to significantly affect Ang-2 or Cys-C levels, such as certain immunosuppressive drugs (e.g., cyclosporine), specific enzyme-inducers (e.g., rifampicin), or enzyme-inhibitors (e.g., cimetidine) within 6 weeks before enrollment.

### 2.2. Data collection

Fasting venous blood (5 mL) was extracted from all patients with ACS in the morning upon admission and placed in a collection tube for natural agglutination at room temperature for 30 minutes. After blood coagulation, the upper serum layer was collected and frozen at −80℃ for later use following centrifugation at 2000 r/min for 20 minutes. Data collected from all participants included age, sex, body mass index, history of diabetes, hypertension, total cholesterol (TC), triglyceride (TG), low-density lipoprotein cholesterol (LDL-C), high-density lipoprotein cholesterol (HDL-C), ACS disease type (ST-segment elevation myocardial infarction, non-ST-segment elevation myocardial infarction, and unstable angina pectoris), and Gensini score. Serum levels of TC, TG, LDL-C, and HDL-C were measured using an automatic biochemical analyzer (Beijing Prang New Technology Co., Ltd.). The Gensini scoring system recommended by the American College of Cardiology was used to evaluate the degree of coronary artery stenosis and the severity of atherosclerosis. The higher the score, the more severe the coronary artery lesions.^[14]^

### 2.3. Enzyme-linked immunosorbent assay (ELISA)

Serum Ang-2 and Cys-C levels were determined according to the manufacturer’s instructions. The Ang-2 enzyme-linked immunosorbent assay (ELISA) kit (HD-10028K) was purchased from Shanghai Luding Biotechnology Co., Ltd., and the Cys-C ELISA kit (D751041-0048) was purchased from Shanghai Jianglai Biotechnology Co., Ltd.. All procedures were performed strictly following the manufacturer’s instructions.

### 2.4. Statistical analysis

Statistical software (SPSS 21.0, SPSS, Inc., Chicago), GraphPad Prism 8.01 Software (GraphPad Software Inc., San Diego), and MedCalc Software Ltd. (MedCalc Software Ltd, Belgium) were used for statistical analysis and data mapping. The Shapiro–Wilk test was used to assess normality, and normally distributed data were represented by mean ± standard deviation. Independent sample *t*-test was used to compare 2 groups. Non-normally distributed data were represented by the median value (minimum value, maximum value), and the Mann–Whitney *U* test was used for analysis. Counting data are expressed in terms of cases and percentages using chi-square tests.

For normally distributed data, the Pearson correlation coefficient was used to analyze the correlation between the indicators, as it can accurately measure the linear relationship between variables. Spearman’s correlation coefficient was used for non-normally distributed measurement data. It is based on the rank of data and can effectively reflect the monotonic relationship between variables without relying on the data distribution form.

Logistic regression was used to determine the independent risk factors for ACS. As the occurrence of ACS is a binary variable (occurrence vs nonoccurrence), the logistic regression model is well-suited for this scenario. It estimates the effect of each independent variable on the dependent variable (ACS occurrence), thereby identifying significant independent risk factors. This approach allows a clear assessment of how each independent variable influences the likelihood of ACS, helping to identify critical risk factors.

ROC analysis was used to analyze the predictive value of serum Ang-2, Cys-C, or Ang-2 combined with Cys-C for the early diagnosis of ACS. The ROC curve displays the relationship between the sensitivity and specificity of a diagnostic test. By calculating the area under the curve (AUC), the diagnostic efficacy of different indicators or combinations of indicators can be quantitatively compared, aiding in evaluating their value in the early diagnosis of ACS. All *P* values were obtained from bilateral tests, and *P* < .05 was considered statistically significant.

## 3. Results

### 3.1. Clinical baseline characteristics of the enrolled population

In this study, 180 patients with ACS were included as participants, and 120 healthy volunteers were included as controls. Statistical analyses were conducted using the baseline clinical data of all participants. As shown in Table 1, no significant differences were observed in age, sex, body mass index, diabetes history, or hypertension history between the patients with ACS and controls (*P* > .05). Significant differences were observed in TC, TG, LDL-C, HDL-C, ACS disease types, and Gensini scores (*P* < .05).

**Table 1:** Clinical baseline data of the enrolled population.

Parameters	Normal group (N = 120)	ACS group (N = 180)	*P* value
Age (yr)	60.40 ± 4.26	59.84 ± 5.15	.323
Gender			
Male (N, %)	72 (60.00%)	102 (56.67%)	.567
Female (N, %)	48 (40.00%)	78 (43.33%)	
BMI (kg/m^2^)	24.46 ± 1.34	24.69 ± 1.33	.153
Diabetes (N, %)	35 (29.17%)	64 (35.56%)	.249
Hypertension (N, %)	30 (25.00%)	52 (28.89%)	.459
TC (mmol/L)	4.27 ± 0.49	4.45 ± 0.64	.010
TG (mmol/L)	1.47 ± 0.28	1.58 ± 0.44	.019
LDL-C (mmol/L)	2.58 ± 0.54	2.73 ± 0.63	.041
HDL-C (mmol/L)	1.22 ± 0.33	1.12 ± 0.43	.043
ACS disease types (N, %)			
STEMI	–	78(43.33%)	< .001
NSTEMI		55(30.56%)	
UAP		48(26.67%)	
Gensini scores	–	53.26 ± 12.47	< .001

The counting data were presented as the number of cases and percentage, and analysed using the chi-square test. The measurement data conforming to the normal distribution were expressed as the mean ± standard deviation, and the comparison between the 2 groups was performed by *t* test. *P* < .05 was considered statistically significant.

BMI = body mass index, HDL-C = high-density lipoprotein cholesterol, LDL-C = low-density lipoprotein cholesterol, NSTEMI = non-ST elevation myocardial infarction, STEMI = ST-segment elevation myocardial infarction, TC = total cholesterol, TG = triglycerides, UAP = unstable angina pectoris.

### 3.2. Serum Ang-2 and Cys-C levels were significantly high in patients with ACS

**Figure 1.**
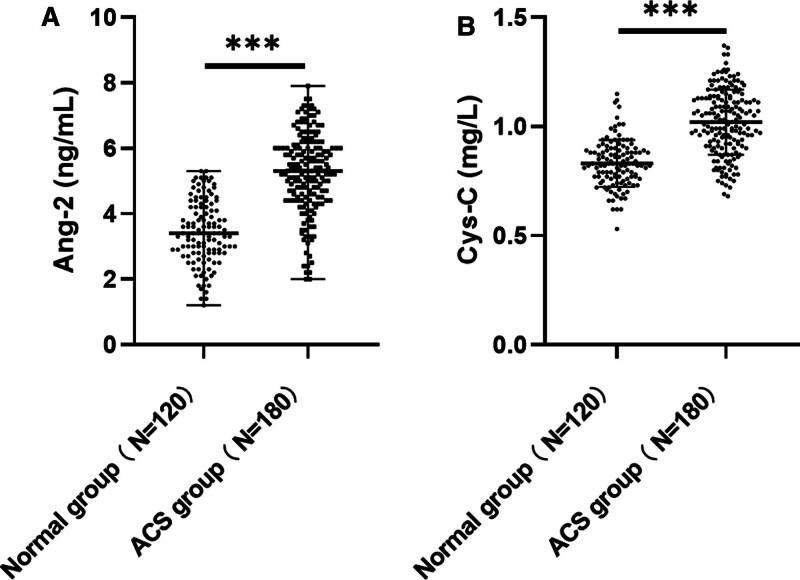
Serum Ang-2 and Cys-C levels were significantly high in patients with ACS. (A) Serum Ang-2 level was determined using ELISA (B) ELISA kit was used to determine the level of Cys-C in serum. The data in (A) were non-normally distributed, represented by median value (minimum value, maximum value) and assessed using the Mann–Whitney *U* test; that in (B) was normally distributed, represented by mean ± standard deviation and assessed using the independent sample *t* test. *** *P* < .001. ACS = acute coronary syndrome, Ang-2 = angiopoietin 2, Cys-C = cystatin C, ELISA = enzyme-linked immunosorbent assay.

Plasma Ang-2 levels are elevated in patients with ACS^[9]^; furthermore, elevated Cys-C levels in patients with ACS are associated with increased mortality.^[12]^ Consequently, we measured serum Ang-2 and Cys-C levels using an ELISA kit; the results indicated that serum Ang-2 and Cys-C levels in patients with ACS were significantly higher than those in normal individuals (*P* < .001; Fig. 1A, B). These findings suggest that serum Ang-2 and Cys-C levels are significantly increased in patients with ACS.

### 3.3. Correlation between serum Ang-2 and Cys-C levels and Gensini scores in patients with ACS

**Figure 2.**
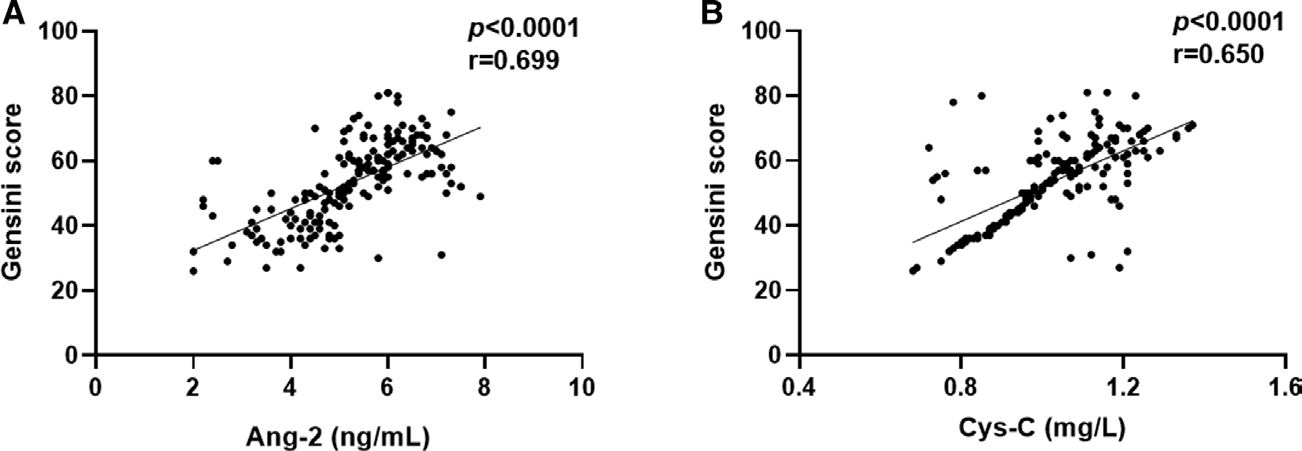
Correlation between serum Ang-2 and Cys-C levels and Gensini scores in patients with ACS. (A) Spearman correlation coefficient was used to analyze the correlation between Ang-2 level and Gensini score in ACS. (B) Pearson correlation coefficient was used to analyze the correlation between serum Cys-C level and Gensini score in ACS. ACS = acute coronary syndrome, Ang-2 = angiopoietin 2, Cys-C = cystatin C.

To evaluate the correlation between serum Ang-2 and Cys-C levels and ACS severity further, Spearman’s or Pearson’s correlation coefficients were used to analyze the correlation between serum Ang-2 and Cys-C levels and Gensini scores in patients, respectively. The analysis revealed a statistically significant positive correlation between serum Ang-2 (*P* < .001, *r* = 0.699, Fig. 2A) and Cys-C (*P* < .001, *r* = 0.650, Fig. 2B) levels and Gensini scores in patients with ACS. These results indicated that serum Ang-2 and Cys-C levels were significantly positively correlated with Gensini scores in patients with ACS.

### 3.4. Independent risk factors for ACS were analyzed using logistic multivariate regression

The occurrence of ACS served as the dependent variable and was coded as 1 for occurrence and 0 for nonoccurrence. The levels of TC, TG, LDL-C, HDL-C, and serum Ang-2 and Cys-C, which were significant at *P* < .05 in Table 1, were selected as independent variables for the subsequent multivariate logistic regression analysis. The results showed that TC, TG, Ang-2, Cys-C, and LDL-C levels were independent risk factors for ACS (Table 2).

**Table 2:** Logistic multivariate regression analysis of ACS occurrence.

Factor	*P*	OR	95% CI
TC (mmol/L)	.044	1.890	1.016–3.517
TG (mmol/L)	.020	2.958	1.186–7.376
LDL-C (mmol/L)	.017	2.130	1.144–3.968
HDL-C (mmol/L)	.464	0.816	0.340–1.960
Ang-2 (ng/mL)	.000	3.125	2.211–4.416
Cys-C × 10^−1^ (mg/L)	.000	2.152	1.573–2.945

ACS = acute coronary syndrome, Ang-2 = angiopoietin 2, CI = confidence interval, Cys-C = cystatin C, OR = odds ratio, HDL-C = high-density lipoprotein cholesterol, LDL-C = low-density lipoprotein cholesterol, TC = total cholesterol, TG = triglycerides.

### 3.5. Serum Ang-2 combined with Cys-C has high diagnostic value in ACS

**Figure 3.**
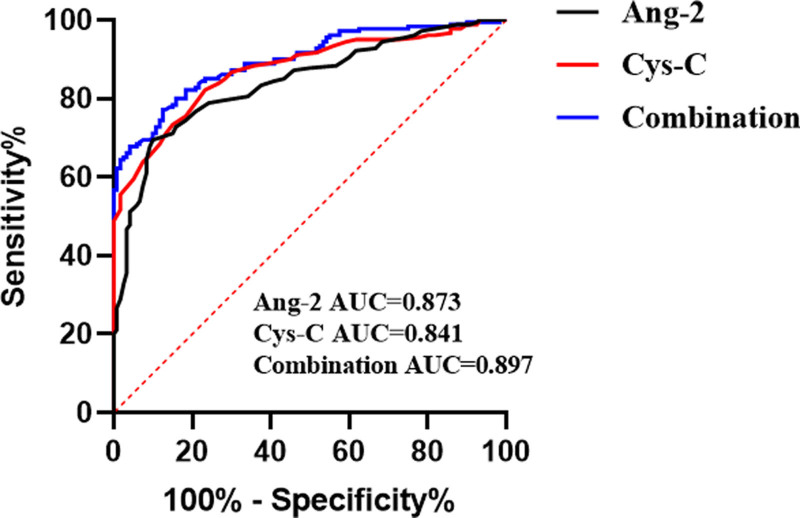
Predictive value of serum Ang-2, Cys-C, or Ang-2 combined with Cys-C for ACS occurrence. ACS = acute coronary syndrome, Ang-2 = Angiopoietin 2, AUC = area under the curve, Cys-C = cystatin C.

Based on the aforementioned findings, an ROC curve analysis was performed to further assess the diagnostic utility of serum Ang-2 and Cys-C levels in patients with ACS. The results showed that the AUC of serum Ang-2 levels for patients with ACS was 0.873, the sensitivity was 82.22%, the specificity was 76.67%, and the cutoff value was 4.250 (Fig. 3). The AUC for serum Cys-C level in patients with ACS was 0.841, with a sensitivity of 69.44%, specificity of 90.00%, and a cutoff value of 0.945 (Fig. 3). The combined AUC of serum Ang-2 and Cys-C levels for patients with ACS was 0.897, with a sensitivity of 77.22%, specificity of 87.50%, and a cutoff value of 0.633 (Fig. 3). Lastly, MedCalc software was used to compare and analyze the AUCs, revealing that the diagnostic performance of serum Ang-2 in combination with Cys-C levels in patients with ACS was significantly superior to that of serum Ang-2 (*P* = .027) or Cys-C levels (*P* = .001) alone (Table 3). These results indicate that serum Ang-2 combined with Cys-C levels has a better diagnostic efficacy in patients with ACS.

**Table 3:** Pairwise comparison of ROC curves.

Pairwise comparison of ROC curves	*P* value
Ang-2 to Cys-C	.221
Ang-2 to combination	.027
Cys-C to combination	.001

Ang-2 = angiopoietin 2, Cys-C = cystatin C, ROC = receiver operating characteristic.

*P *< .05 was considered statistically significant.

## 4. Discussion

ACS is a type of coronary artery disease. The main pathological basis is the rupture or erosion of unstable coronary atherosclerotic plaques, leading to thrombosis, which disrupts the coronary blood flow and causes myocardial ischemia. Patients often present with symptoms of discomfort, such as chest pain, chest tightness, and palpitations. Their quality of life is affected.^[14,15]^ Currently, cTnT is used as a biomarker for the clinical diagnosis of ACS. However, other diseases such as chronic renal failure, cerebrovascular accidents, acute pulmonary embolism, hypertensive crisis, tachy- or bradyarrhythmia, acute inflammatory myocarditis, and skeletal myopathies can also lead to elevated levels of these markers.^[16]^ In addition, myocardial troponin is not consistently elevated in acute myocardial infarction, and due to its delayed release into the bloodstream, there is a lack of sensitivity within the first few hours, leading to a “troponin-blind period.”^[17]^ Currently, there is a lack of reliable and simple screening methods for ACS in clinical settings. Early diagnosis can effectively delay the progression of ACS and improve patient prognosis.

In a large community-based study, higher Ang-2 concentrations were associated with a higher risk of all-cause mortality and cardiovascular death.^[18]^ Ischemia and hypoxia environment may stimulate the release of Ang-2 by endothelial cells, reduce vascular stability, and promote endothelial cell activation, new angiogenesis, and remodeling.^[19,20]^ Ang-2 is highly expressed in ischemic myocardium, with an expression level higher than that of other classical angiogenesis factors.^[8]^ Free radicals are generated during Cys-C oxidation, which increases the formation of foam cells and induces arterial lumen stenosis, resulting in arterial lumen stenosis.^[13]^ Cys-C can effectively reflect the severity of arterial vessel damage in ACS.^[21]^ The results of this study showed that serum levels of Ang-2 and Cys-C were significantly increased in patients with ACS. In addition, the Gensini score fully considers the number, location, and degree of stenosis of coronary artery lesions and is widely used in the clinical quantitative analysis of coronary artery lesions, enabling the quick assessment of coronary artery lesions, identification of high-risk patients, and provision of timely treatment.^[22]^ In this study, the Gensini score was used to assess the severity of ACS, and the results showed that serum Ang-2 and Cys-C levels in patients with ACS were significantly positively correlated with Gensini scores, which was consistent with previously reported results.^[21]^ These findings suggest that Ang-2 and Cys-C may promote the occurrence and development of ACS.

The value of Ang-2 in predicting cardiovascular adverse events (MACE) has been widely reported, and Ang-2 is an independent predictor of MACE or all-cause mortality in patients with chronic kidney disease.^[23]^ In adults without cardiovascular disease at baseline, Ang-2 is associated with heart failure or death events, and in individuals with preexisting heart failure, Ang-2 is associated with disease severity.^[24]^ The level of Ang-2 may be an indicator of accelerated atherosclerosis.^[25]^ Eiryu Sai et al^[26]^ found that elevated levels of Cys-C are a predictor of recurrence of cardiovascular events, possibly because Cys-C contributes to the pathophysiological processes of atherosclerosis and inflammation. Serum Cys-C level is an independent risk factor for heart failure in older populations.^[27]^ In addition, Cys-C is an independent predictor of cardiac adverse events in patients with ACS^[13]^; however, to date, the predictive value of Ang-2 and Cys-C in the early diagnosis of ACS has not been reported. In this study, logistic regression analysis of multiple factors showed that elevated Ang-2 and Cys-C levels were independent risk factors for ACS. The ROC curve was used to evaluate the efficacy of Ang-2 and Cys-C in the early diagnosis of ACS. The AUC of the combined serum Ang-2 and Cys-C detection was 0.897, which was significantly higher than that of serum Ang-2 (AUC 0.873) or Cys-C (AUC 0.841) alone. A MedCalc comparative analysis verified that the combined detection of serum Ang-2 and Cys-C can significantly improve the diagnostic efficiency of ACS and has an important clinical reference value for the early diagnosis of ACS.

To highlight the advantages of the combined Ang-2 and Cys-C detection further, we compared its diagnostic performance with that of traditional methods. Electrocardiography is the first-line tool for diagnosing ACS. However, it only reflects the electrical activity of the heart at a specific moment. Repeated tests are required when a patient’s condition changes, and waveform interpretation depends on the subjective judgment of the doctor, which can lead to interpretive discrepancies. The sensitivity of the standard 12-lead electrocardiography for diagnosing ACS is approximately 40.5%, whereas its specificity is 95%.^[28]^ cTnT, another commonly used marker, has an overall sensitivity of 57% and a specificity of 71% for diagnosing ACS.^[29]^ However, cTnT levels may also increase in non-ACS conditions, affecting diagnostic accuracy.^[30,31]^ CK-MB, a frequently utilized cardiac enzyme, has a sensitivity of approximately 30% for early ACS diagnosis, although its specificity is as high as 100%.^[32]^ CK-MB levels typically do not increase within the first 3 hours after symptom onset. They may also increase in noncardiac conditions such as skeletal muscle injury,^[33]^ leading to false-positive diagnoses. In contrast, the combination of Ang-2 and Cys-C demonstrated a more balanced performance in terms of sensitivity and specificity, with a higher AUC. This makes it promising for the early diagnosis of ACS and provides additional valuable information that complements existing traditional diagnostic methods.

In summary, this study confirmed that serum levels of Ang-2 and Cys-C are elevated in patients with ACS, and the combined detection of these 2 biomarkers exhibits high diagnostic efficacy for ACS. This finding has the potential to provide a valuable reference for the early diagnosis, prevention, and treatment of ACS. Nevertheless, this study had some limitations. First, the sample size was relatively small, and the study involved a single-center retrospective analysis. The single-center design and relatively small sample size may have led to a selection bias. The characteristics of the patients in our hospital may not be representative of those in other regions. For example, different regions may have variations in genetic backgrounds, lifestyles, and environmental factors, which could potentially influence Ang-2 and Cys-C levels and their relationships with ACS. Moreover, the small sample size may have limited the statistical power, potentially leading to missed subtle associations. Therefore, the results of this study may not be generalizable to other populations. To further strengthen the conclusions and improve the reliability of the results, future research should involve expanded sample sizes and multicenter prospective studies to elucidate the early diagnostic value of Ang-2 and Cys-C levels in ACS comprehensively.

## Acknowledgments

We thank all the researchers who participated in data processing, we would like to thank Editage (www.editage.cn) for English language editing.

## Author contributions

**Conceptualization:** Qian Zou.

**Data curation:** Qian Zou, Yuxia Huang, Yabin Peng, Feilin Ye.

**Formal analysis:** Qian Zou, Yuxia Huang, Xuanhao Lu, Jie Xu, Ling Fang.

**Investigation:** Xuanhao Lu.

**Methodology:** Qian Zou.

**Project administration:** Yabin Peng, Jie Xu.

**Resources:** Qian Zou, Yabin Peng, Xuanhao Lu, Ling Fang.

**Software:** Qian Zou, Jie Xu, Ling Fang.

**Supervision:** Qian Zou, Yuxia Huang, Ling Fang.

**Validation:** Qian Zou, Yuxia Huang, Yabin Peng, Xuanhao Lu, Feilin Ye, Jie Xu, Ling Fang.

**Visualization:** Yabin Peng, Feilin Ye.

**Writing** – **original draft:** Qian Zou.

**Writing** – **review & editing:** Ling Fang.
